# Choice of Treatment Modality and Validity of Direct Surgery for Complex Posterior Inferior Cerebellar Artery-Related Aneurysms

**DOI:** 10.3390/jcm14238270

**Published:** 2025-11-21

**Authors:** Fumihiro Hamada, Hitoshi Fukuda, Naoki Fukui, Yusuke Ueba, Motonobu Nonaka, Mitsuhiro Takemura, Namito Kida, Tetsuya Ueba

**Affiliations:** Department of Neurosurgery, Kochi Medical School, Kochi University, Nankoku 783-8505, Japan; hamada-fumihiro@kochi-u.ac.jp (F.H.); naofukui@kochi-u.ac.jp (N.F.); yueba@kochi-u.ac.jp (Y.U.); mtb.nonaka@gmail.com (M.N.); light.extent@gmail.com (M.T.); jm-kidanamito@kochi-u.ac.jp (N.K.); tueba@kochi-u.ac.jp (T.U.)

**Keywords:** posterior inferior cerebellar artery aneurysm, occipital artery-posterior inferior cerebellar artery bypass, transcondylar fossa approach, antiplatelet therapy, hybrid treatment

## Abstract

**Background/Objectives:** Complex aneurysms of the posterior inferior cerebellar artery (PICA) remain challenging because of their deep location, variable morphology, and proximity to critical neurovascular structures. Although endovascular therapy is preferred, its feasibility is limited in wide-necked, fusiform, or dissecting lesions. We describe our tertiary referral hospital single-center experience with tailored microsurgical and endovascular strategies—emphasizing occipital artery–PICA (OA-PICA) bypass, transcondylar fossa craniotomy, and cerebellomedullary fissure opening—and analyze perioperative factors that influence outcome. **Methods:** All consecutive patients treated for PICA origin or distal-PICA aneurysms between January 2021 and April 2025 were retrospectively reviewed. Demographics, aneurysm characteristics, procedure type, antithrombotic regimen, complications, diffusion-weighted MRI findings, and 3-month modified Rankin Scale scores were collected. **Results:** Twelve aneurysms (mean age 61.4 ± 15.2 years; 8 women) were treated: trapping + OA-PICA bypass in 5, direct clipping in 2, flow diverter in 1, endovascular parent artery occlusion in 2, coil embolization in 1, and a hybrid bypass-plus-coil strategy in 1. Two cases were ruptured aneurysms. Perioperative aspirin was used in 2/5 bypass cases; heparin was added in one hybrid case. Asymptomatic PICA-territory infarcts occurred in the three bypasses performed without antiplatelet therapy (one with intra-anastomotic thrombus). No leaks or subcutaneous collections of cerebrospinal fluid were encountered, and no graft occlusions were observed. At 3 months, 9/12 patients achieved a good outcome (mRS 0–2); among them, only one patient with subarachnoid hemorrhage (SAH) experienced postoperative worsening of the mRS. Two cranial nerve palsies (one permanent, one transient) and one wound site hematoma (heparin-associated) resolved without sequelae. **Conclusions:** Meticulous operative planning allows safe treatment of complex PICA aneurysms. Perioperative aspirin appears beneficial for OA-PICA bypass, whereas perioperative heparin increases bleeding risk. Individualized selection of endovascular, microsurgical, or combined strategies yields favorable early neurological outcomes in this demanding subset of cerebrovascular disease.

## 1. Introduction

Intracranial aneurysms involving the posterior inferior cerebellar artery (PICA) are rare, yet pose unique therapeutic challenges. In particular, the morphology of PICA-related aneurysms is often complex, such as wide-necked, fusiform, or dissecting, which makes simple intrasaccular coiling less feasible [1,2,3,4]. In such situations, microsurgical approaches—such as direct clipping, trapping with or without occipital artery–PICA bypass—become indispensable, each requiring meticulous dissection around the lower cranial nerves and brainstem, precise clip application, and careful bypass construction to preserve distal flow [5,6]. Recent advances in skull base exposure, such as condylar fossa drilling, along with progress in intradural techniques, particularly the systematic unilateral opening of the cerebellomedullary fissure (CMF), further improved the safety and efficacy of these procedures [7,8,9]. In this study, we detail our experience with 12 consecutive complex PICA aneurysms, emphasizing the surgical strategies, intraoperative decision-making, which underpin successful management of this formidable vascular pathology.

## 2. Materials and Methods

We performed a single-center, retrospective review of consecutive patients who underwent treatment for aneurysms located anywhere from the origin of the PICA to its distal segments at Kochi University Hospital between 1 January 2021 and 30 April 2025. The choice of the treatment period was based on reorganization of our treatment protocols of complex intracranial aneurysms when flow diverters became available in our facility at the end of the year 2020. Both ruptured and unruptured aneurysms were eligible for inclusion, and no patients were excluded, yielding a study cohort of 12 cases. The institutional review board approved the protocol (approval No. ERB-111860); the need for written informed consent was waived because only de-identified data were analyzed. For each patient we extracted demographic variables, aneurysm characteristics (segmental location, maximum diameter, morphology). Procedural details and perioperative complications were gathered from operative notes, anesthesia records, and the electronic medical record. Follow-up imaging was routinely performed as a clinical practice to assess aneurysm occlusion and vessel patency, and the results were retrospectively extracted from medical records for this analysis.

### 2.1. Strategic Choice of Endovascular or Open Surgical Management

**Figure 1:**
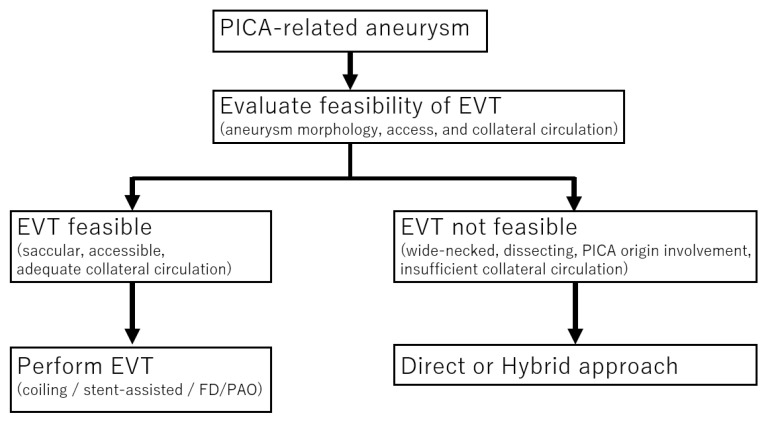
Simplified treatment decision flow for PICA-related aneurysms. EVT: Endovascular treatment.

At our institution, the treatment paradigm for PICA-related aneurysms is structured to favor endovascular therapy as the primary modality. Endovascular techniques include simple coil embolization, balloon-assisted or stent-assisted coiling, the deployment of flow-diverting stents, and, in cases where collateral circulation is sufficient, parent-artery occlusion. For elective endovascular procedures, aspirin and clopidogrel (dual antiplatelet agents) were given preoperatively and perioperatively, according to our institutional protocol. Microsurgical intervention is reserved for situations in which specific anatomical or clinical factors necessitate an open approach, such as aneurysms with a wide-neck configuration (defined as a neck diameter exceeding 4 mm or a dome-to-neck ratio below 1.5), fusiform or dissecting morphologies requiring vessel reconstruction, involvement of the PICA origin that precludes safe endovascular treatment, contraindications to dual antiplatelet therapy, or when endovascular embolization is unsuccessful or incomplete. When surgery is indicated, the approaches employed consist of direct clipping via a transcondylar fossa approach, trapping combined with an OA–PICA bypass to maintain distal perfusion, and hybrid procedures in which intraoperative coil occlusion is performed immediately following the revascularization procedure within the same surgical session. For surgical and hybrid procedures, antiplatelet agents were administered at the discretion of the treating physician. In general, aspirin administration was considered when the surgical procedure included OA-PICA bypass or was combined with coil embolization. All treatment strategies are determined through consensus in a dedicated multidisciplinary cerebrovascular conference, ensuring that each patient receives an individualized management plan based on anatomical considerations, clinical presentation, and the best available evidence. The treatment decision flow is shown in Figure 1.

### 2.2. Operative Technique

The patient was positioned in a semi-prone park-bench position, and a hockey stick–shaped skin incision was made. In cases requiring OA-PICA bypass, the OA was harvested by making a skin incision and subcutaneous dissection from the parietal side. Proximal to the splenius capitis, the muscle was incised along the OA, together with the longissimus capitis, and dissection was carried out down to the digastric groove. The OA was then mobilized laterally and preserved in situ to maintain blood flow until needed. The remaining suboccipital muscles were reflected en bloc laterocaudally with the musculocutaneous flap. The C1 posterior arch was generally preserved; however, in cases with a low-lying caudal loop of the PICA or tonsillar descent, the C1 posterior arch was also removed. The posterolateral portion of the foramen magnum was resected. After coagulating and dividing the posterior condylar emissary vein, the posterior condylar fossa was exposed. The lateral extent of the craniotomy was carefully planned based on preoperative CT imaging with volume rendering reconstruction using a workstation—ZIO STATION (Ziosoft Inc., Tokyo, Japan) to visualize the medial extension of the mastoid air cells, and was deliberately limited to avoid opening the mastoid cells. When a posterior condylar emissary vein was present, the extracranial portion of the posterior condylar canal was drilled, allowing the posterior condylar emissary vein to be mobilized from within the posterior condylar canal to the outside. Subsequently, the posterior condylar emissary vein was coagulated and retracted up to its junction with the sigmoid sinus. Using the foramen magnum, medial edge of the sigmoid sinus, and occipital condyle as landmarks, the posterior half of the jugular tubercle was drilled down to the level of the hypoglossal canal. This completed the transcondylar fossa approach. The dura was opened, followed by unilateral opening of the CMF and the cerebellum was gently retracted in a rostrodorsal direction to establish the operative field. After unilateral opening of the CMF, the arachnoid strands anchoring cranial nerve (CN) IX are carefully detached to extend the dissection into the cerebellomedullary cistern. The lateral pontomedullary membrane, which demarcates the posterior condylar canal from the cerebellopontine cistern, lies between cranial nerves IX and VIII [10]. Incising the arachnoid just rostral to the CN IX therefore unites the CMF with the cerebellomedullary cistern, providing a substantially wider operative corridor. Once adequate mobilization of the cerebellum was achieved, gentle elevation of the cerebellar tonsil provided a panoramic view of the V4 segment, proximal PICA, and lower cranial nerves. In cases requiring OA-PICA bypass, this approach generally allowed sufficient exposure for anastomosis even when the caudal loop of the PICA was situated relatively high. Clipping or trapping of the aneurysm was then performed as appropriate. Regardless of the presence or absence of a bypass graft, the dura was reconstructed using collagen matrix dural substitutes (DuraGen^®^, Integra LifeSciences, Tokyo, Japan).

### 2.3. Outcome Assessment

Clinical outcome was assessed at 3 months after the procedure using the modified Rankin Scale (mRS) by an independent neurosurgeon. A good outcome was defined as mRS 0–2, and a poor outcome as mRS 3–6. New focal neurological deficits occurring within 30 days of the procedure were recorded. Complications were assessed based on the presence of neurological deficits, lesions on diffusion-weighted imaging (DWI), intracranial hemorrhage, or cerebrospinal fluid leak.

## 3. Results

Clinical characteristics of 12 patients with PICA-related aneurysms are summarized in Table 1. We primarily consider endovascular therapy as the first-line treatment for PICA-related aneurysms; however, endovascular procedures were performed in only 4 of the 12 cases (No. 4, 5, 11, and 12). The PICA aneurysms in our cohort included seven saccular, one fusiform, and four dissecting aneurysms. All the saccular aneurysms met the predefined criteria for a wide neck (dome-to-neck ratio < 1.5 or neck > 4 mm), which means all the aneurysms in our cohort had either of wide-necked, fusiform, or dissecting morphology. We believe this affected the treatment choice of our cohort, where only one aneurysm was treated with simple intrasaccular coil embolization while the others were treated with surgical clipping, parent artery occlusion with or without bypass, or a flow diverter. This may also reflect that PICA-related aneurysms have complex morphologies in nature. Case No. 4 involved a giant vertebral artery (VA) aneurysm including the PICA. Although bypass surgery would have been the radical treatment, flow diverter placement was prioritized because of the patient’s advanced age and preoperative comorbidities. In Cases No. 5 and 12, sufficient collateral circulation allowed for parent artery occlusion. Case 11 was a patient who presented with recurrence of an aneurysm 21 years after clipping, accompanied by gait disturbance due to the mass effect of the aneurysm on the medulla. Coil embolization was successfully performed while preserving the PICA. The mRS score was 4, but there was no deterioration compared with the preoperative status. Case No. 6 was a dissecting fusiform aneurysm involving the PICA, treated with bypass and trapping. Case No. 7 was a thrombosed distal PICA aneurysm with poor collateral supply, for which bypass and trapping were also performed. Case No. 8 was a basilar artery (BA)–anterior inferior cerebellar artery (AICA) aneurysm, which was included in this study because the AICA formed an AICA–PICA common trunk. Because the AICA arose from the aneurysm neck, preservation was difficult; therefore, an OA–AICA bypass, analogous to an OA–PICA bypass, was performed, followed by coil embolization as a hybrid approach. In Case No. 9, clipping was selected due to an allergy to antiplatelet agents.

### Illustrative Cases

Case No. 9

**Figure 2:**
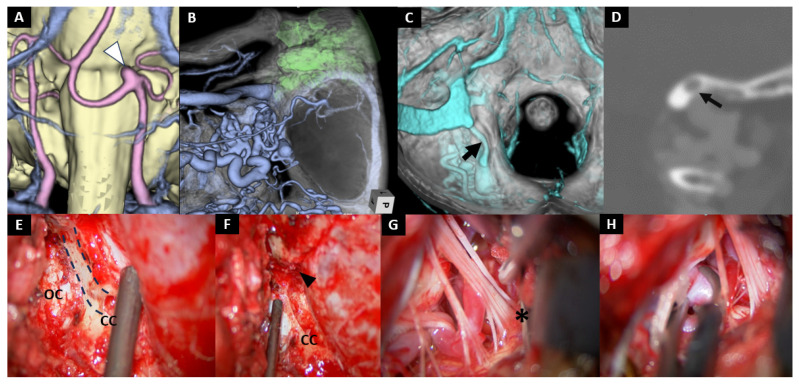
Case 9: Left transcondylar fossa approach for an VA-PICA aneurysm. (**A**) Volume rendering was performed on T1-weighted thin-slice images including the brainstem, and the dataset was fused with magnetic resonance angiography (MRA) on a workstation to delineate the location of the aneurysm. The aneurysm (white arrowhead) is located at the left pontomedullary junction. (**B**) 3D-reconstructed CT angiogram shows preoperative assessment of the anatomical relationship among the mastoid air cells, digastric groove, and sigmoid sinus. (**C**) Reconstructed 3D-CT angiogram identifies the course of the condylar emissary vein (arrow). (**D**) Coronal section of the CT angiogram shows that a well-developed condylar emissary vein makes outer cortical bone thin (arrow). (**E**–**H**) Intraoperative photographs. (**E**) The outer wall of the condylar canal (dotted line) is removed with a diamond burr. (**F**) The condylar canal is opened by coagulating and collapsing the condylar emissary vein up to the posterior margin of the sigmoid sinus (arrowhead). (**G**) Dissection of the arachnoid membrane around the root origin of the CN IX (*) allows mobilization of the cerebellum. (**H**) The cerebellum is gently elevated with a spatula to obtain the operative corridor, followed by aneurysm clipping. OC: occipital condyle; CC: condylar canal; SS: sigmoid sinus.

A 70-year-old woman was under monitoring for an unruptured pericallosal artery aneurysm when a de novo VA–PICA aneurysm was incidentally detected (Figure 2A). She was referred to our hospital for treatment. Although stent-assisted coil embolization was initially scheduled, it had to be abandoned due to an allergic reaction to antiplatelet agents, leading us to opt for microsurgical clipping. Preoperative planning involved simulating the extent of mastoid air cell development, and the craniotomy was carefully tailored to remain within a range that would not open the mastoid air cells (Figure 2B,C). Intraoperatively, a markedly developed condylar emissary vein made the condylar canal enlarged, resulting in thinning of its caudal bony floor (Figure 2D,E). This anatomical feature allowed us to easily drill away the floor, opening the condylar canal. By doing so, we could gently exteriorize the emissary vein without causing significant bleeding, and then coagulate and shrink it up to its confluence with the sigmoid sinus (Figure 2F). Once the emissary vein was managed, the opened condylar canal was clearly visualized, and the condylar fossa was efficiently removed using rongeurs. This step revealed critical anatomical landmarks necessary for subsequent jugular tubercle resection, including the medial edge of the sigmoid sinus, the foramen magnum, and the occipital condyle. Together, these landmarks provided a clear orientation for safe drilling and exposure. A transcondylar fossa approach was then performed, followed by meticulous opening of the CMF. Thorough dissection was carried out up to the root entry zone of the CN Ⅸ (Ⅸ: * Figure 2G) to secure sufficient mobility of the cerebellum and to optimize the operative corridor. This maneuver allowed safe visualization and clipping of the aneurysm (Figure 2H).

Case No. 10

**Figure 3:**
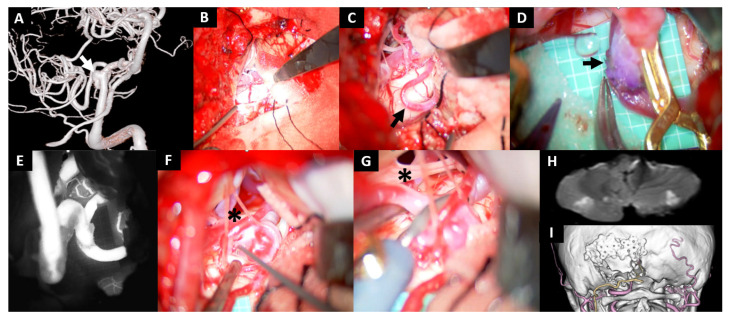
Case 10: A left VA–PICA aneurysm treated with OA–PICA bypass and trapping via the transcondylar fossa approach. (**A**) 3D-rotational digital subtraction left vertebral angiogram shows a small PICA aneurysm with a wide neck (arrow). (**B**–**G**) Intraoperative photographs. (**B**) Retraction of the cerebellum alone does not expose the distal PICA. (**C**) Opening the cerebellomedullary fissure allows wide exposure of the caudal loop of the PICA (arrow). (**D**) Intraluminal white thrombus is formed during the anastomosis (arrow) but resolved with gentle massage. (**E**) Patency of the bypass is confirmed by indocyanine green angiography. (**F**,**G**) The aneurysm and the origin of PICA is adjacent to the CN XII (*). Gentle retraction of the nerve by the assistant enables trapping with aneurysmal clips. (**H**) Postoperative MRI diffusion-weighted imaging shows ischemic changes in bilateral PICA region. (**I**) Postoperative 3D-reconstructed CT angiogram shows disappearance of the aneurysm and good patency of the OA-PICA bypass (yellow).Extracranial arteries unrelated to the bypass are indicated in pink.

A 57-year-old woman with a history of subarachnoid hemorrhage caused by a ruptured distal anterior cerebral artery aneurysm was referred to our institution for treatment when a newly developed aneurysm was detected at the left VA-PICA bifurcation. Given the extremely small dome-to-neck ratio and the difficulty in preserving the PICA, we performed an OA-PICA bypass and trapping procedure (Figure 3A). No antiplatelet agents were administered during the perioperative period. Upon craniotomy, gentle elevation of the cerebellum did not allow visualization of the caudal loop of the PICA (Figure 3B). By thoroughly opening the CMF and adjusting the viewing angle to look upward from the dorsal side, we were able to expose the PICA by carefully drawing it into the operative field (Figure 3C, arrow). During additional suturing for blood leak from the anastomosis site after declamp, a whitish thrombus was observed (Figure 3D, arrow). After completion of the anastomosis, indocyanine green angiography confirmed satisfactory bypass flow (Figure 3E). The CN Ⅻ was found adherent to the cranial side of the aneurysmal dome (Figure 3F, asterisk). Trapping was performed while the assistant retracted the CN XII (Figure 3G, asterisk). Postoperatively, although the patient remained asymptomatic, a small infarction was noted on MRI DWI, presumably due to embolism from the formed thrombus (Figure 3H). CT angiography demonstrated disappearance of the aneurysm and a patent bypass (Figure 3I), and the patient was discharged home without any neurological deficits.

Case No. 8

**Figure 4:**
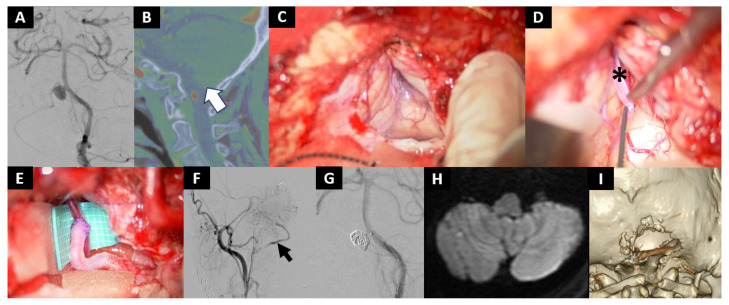
Case 8: A right BA–AICA aneurysm treated by a hybrid strategy combining bypass reconstruction and coil embolization. (**A**) Digital subtraction left vertebral angiogram shows right BA–AICA aneurysm with an AICA–PICA variant originating from the neck of the aneurysm. (**B**) Sagittal section of the CT angiogram shows that the distal segment of the AICA-PICA courses caudal to the tonsil (white arrow). (**C**–**G**) Intraoperative photographs obtained during the combined bypass reconstruction and coil embolization in the hybrid operating room. (**C**) A limited craniotomy is made below and medial to the inferior nuchal line. The mastoid air cell is not opened. (**D**,**E**) The bypass was performed at the tonsillar segment of the AICA-PICA (*) without retraction of the cerebellum. (**F**) Intraoperative digital subtraction left vertebral angiogram confirms good bypass patency (black arrow). (**G**) Subsequently, the aneurysm and the origin of AICA are embolized with coils. (**H**) Postoperative MRI diffusion-weighted imaging shows no ischemic infarction. (**I**) 3D-reconstructed plain CT confirms that a small craniotomy is performed as planned and the mastoid air cell is not opened.

A 67-year-old man was referred to our hospital for an unruptured BA–AICA aneurysm, which enlarged from 4 mm to 7 mm over time (Figure 4A). The AICA formed a common trunk with the PICA, which ultimately reached the cerebellar tonsil (Figure 4B). Stent-assisted coil embolization was not considered feasible, because the AICA originated from the neck of the aneurysm. Also, direct clipping of the aneurysm was considered challenging because of midline location of the aneurysm and the presence of adjacent cranial nerves. Thus, we took a hybrid endovascular and surgical strategy, where the AICA was reconstructed with bypass and both the aneurysm and the origin of the AICA were embolized.

The patient received aspirin 100 mg during the perioperative period. The craniotomy was limited to the area caudal to the inferior nuchal line (smaller than usual) to reduce the risk of hemorrhagic complications associated with systemic heparinization at the following endovascular procedure and to avoid opening of the mastoid air cells. (Figure 4C). The planned recipient vessel, the PICA segment of the AICA on the caudal side of the tonsil, was identified without difficulty (asterisk, Figure 4D). The anastomosis was completed uneventfully (Figure 4E). Systemic heparinization was initiated immediately after the OA–PICA bypass, and coil embolization was performed. The bypass demonstrated good patency (arrow), and coil embolization of the aneurysm and the origin of AICA was completed without complications (Figure 4F,G). However, immediately after skin closure, continuous bleeding from the wound was observed, which was subsequently resolved by reopening the wound and meticulous hemostasis of the muscles and subcutaneous tissues. Postoperative MRI revealed no ischemic lesions (Figure 4H). Postoperative 3D-reconstructed plain CT confirms a small craniotomy as planned (Figure 4I). The patient was discharged without any neurological deficits.

## 4. Discussion

This study reviewed our institutional experience with 12 consecutive complex PICA aneurysms treated by endovascular, microsurgical, or hybrid approaches. Although small in number, our series provides insights into both clinical outcomes and technical considerations of these challenging lesions.

### 4.1. Clinical Outcomes

PICA-related aneurysms are relatively rare [11], and their surgical management is particularly demanding because of their deep and narrow location and adjacency to critical structures such as the lower cranial nerves, medulla oblongata, and cerebellum [12]. Because of this inherent complexity, endovascular therapy is generally prioritized as the first-line approach [13]. However, endovascular treatment is occasionally limited in practice due to wide-necked, fusiform, or dissecting morphologies, which hampers safe and durable exclusion of the aneurysm [2]. Although these lesions often require direct microsurgical intervention, most neurosurgeons have only limited exposure to such rare cases [5]. Accordingly, the management of VA–PICA aneurysms requires careful case selection and a multidisciplinary strategy, along with dedicated efforts to maintain surgical expertise including OA–PICA bypass and skull base approaches despite the infrequency of operative opportunities.

Despite the complexity of the lesions, overall treatment outcomes were favorable in our case series with individualized treatment selection strategy. Although intrasaccular coil embolization was performed in only one case, endovascular therapy contributed to safe and effective treatment differently, by parent artery occlusion, branch occlusion, or flow diversion in four cases. Eight of twelve cases required surgical treatment, including a case treated with hybrid strategy, which suggests surgical option still plays a pivotal role to treat complex PICA-related aneurysms. The overall result of the surgical treatment was generally favorable, where one permanent dysphasia in one case of ruptured aneurysm and asymptomatic cerebellar infarction in three cases. The OA-PICA bypass was successful in all five cases and prevented symptomatic cerebellar ischemia associated with therapeutic occlusion of the parent or branch artery.

### 4.2. Technical Considerations

In the following sections, we outline important considerations for direct surgical intervention, focusing on perioperative management, craniotomy, and intradural procedures, which are essential to obtain better overall outcomes of complex PICA-related aneurysms.

Various approaches to access the ventral region from the lateral margin of the foramen magnum have been reported to date. One of the fundamental concepts was proposed by Heros, who introduced the lateral approach [14], emphasizing an upward view from a dorsolateral direction utilizing the caudal aspect of the lower cranial nerves as the surgical corridor. Subsequently, approaches emphasizing the lateral view, such as the far lateral approach and extreme lateral approach, were described [15,16]. Meanwhile, following the dorsolateral perspective advocated by Heros, Bertalanffy et al. reported the dorsolateral suboccipital transcondylar approach [17]. Furthermore, Matsushima et al. modified the transcondylar approach by preserving the condyle while resecting the condylar fossa and the posterior portion of the jugular tubercle, which they termed the transcondylar fossa approach [18]. This was later established, in combination with an intradural unilateral CMF approach, as an effective technique for managing PICA-related aneurysms.

#### 4.2.1. Perioperative Antiplatelet Therapy

When performing an OA-PICA bypass, the use of antiplatelet agents becomes a key issue. To date, very few studies have specifically investigated perioperative antiplatelet therapy in OA–PICA bypass. Although the necessity of dual antiplatelet therapy remains controversial, most reports describe administering at least some form of antiplatelet agent during the perioperative period [5,19,20]. In our present series, among five patients who underwent OA–PICA bypass, asymptomatic ischemic lesions in the PICA territory were observed exclusively in the three patients who did not receive perioperative antiplatelet therapy, suggesting that such therapy should be recommended in this context. In superficial temporal artery–middle cerebral artery (STA-MCA) bypass surgery, antiplatelet therapy has been reported not to increase perioperative complications and to improve clinical outcomes [21], which likely accounts for its widespread use in OA–PICA bypass and other revascularization procedures. Particularly in moyamoya disease, the efficacy of antiplatelet therapy after STA–MCA bypass has been well documented [22]. Kanamori et al. further demonstrated that aspirin suppresses the occurrence of white thrombi at the anastomotic site, whereas heparin increases the risk of hemorrhagic complications in STA–MCA bypass for moyamoya disease [23]. Consistent with these findings, in our series, all three cases performed without aspirin showed DWI-positive lesions—including one case (Case 10) with actual thrombus formation at the anastomosis—and the patient who received heparin (Case 8) developed wound-site bleeding. Although the underlying pathology differs from moyamoya disease, these results collectively suggest that perioperative aspirin should be recommended even in OA–PICA bypass, whereas perioperative heparin may pose an increased bleeding risk.

#### 4.2.2. Craniotomy

The transcondylar fossa approach has been thoroughly described in numerous previous reports [7,24,25], and a detailed technical account is beyond the scope of this paper. One of the refinements we have adopted is the meticulous preoperative simulation of the craniotomy extent. To avoid opening the mastoid air cells that increases the risk of cerebrospinal fluid leakage, we limited the lateral extent of the craniotomy. This can be reliably assessed preoperatively by generating volume rendering images from CT angiography data using a workstation. For evaluating the medial extension of the mastoid air cells, the digastric groove serves as a dependable anatomical landmark, minimizing intraoperative ambiguity. In our experience, the mastoid air cells are not present inferior to the inferior nuchal line. Thus, violation of the mastoid air cells was avoided as long as the craniotomy was limited inferior to the inferior nuchal line. The concept of not opening the mastoid air cells was applied to Case 9, where transpetrosal approach with wide opening of the mastoid air cells for direct clipping of the aneurysm was replaced by combination of endovascular embolization and OA-PICA bypass under a small craniotomy without opening the mastoid air cells.

**Figure 5:**
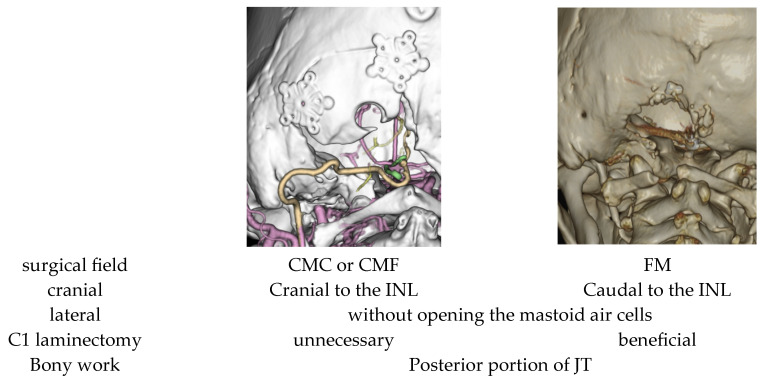
Selection of the Craniotomy extent for PICA related Aneurysms. CMC: cerebellomedullary cistern; CMF: cerebellomedullary fissure; FM: foramen magnum; INL: inferior nuchal line: JT: jugular tubercle.

For OA–PICA bypass, the caudal loop of the PICA—a portion of the tonsillomedullary segment—is generally regarded as the preferred recipient [26,27,28]. In our case as well, the anastomosis was performed on the tonsillomedullary segment. However, cases in which the caudal loop is unsuitable because of dissection or cannot be used owing to anatomic variation are not uncommon [29]. Abe et al. reported that in patients with a high-lying tonsillomedullary segment, a unilateral trans–CMF approach can provide an adequate operative corridor [30]. Moreover, when a bypass must be constructed to the lateral medullary segment or other proximal PICA portions, extending the craniotomy laterally in accordance with the far-lateral approach is considered effective [14,31,32,33]. Fukuda et al. further demonstrated that a far-lateral approach provides, on average, a 13.5 ± 2.2 mm wider working corridor than a traditional midline foramen magnum craniotomy, thereby facilitating access to alternative recipient sites such as the proximal PICA (lateral medullary/p1–p2 region) [34]. To facilitate exposure of and bypass to the proximal PICA, a far-lateral craniotomy with drilling of the posterior one-third or more of the occipital condyle has also been advocated [35]. In such circumstances, a purely medial–caudal corridor is often insufficient, and the bony exposure may ultimately need to extend toward the mastoid region. Regarding the cranial extent, a wider craniotomy generally facilitates placement of brain retractors and elevation of the cerebellum. However, when the surgical field is confined to the caudal end of the tonsil, cerebellar manipulation is seldom required. In such cases, as demonstrated in Case 8, limiting the cranial extent of the craniotomy to the inferior nuchal line was sufficient. Conversely, when the operative field extends more caudally, a C1 laminectomy can significantly improve surgical maneuverability. Although several authors have reported that a C1 laminectomy is not required in the transcondylar fossa approach for PICA aneurysms [5,24], its necessity has not been clearly established [36,37]. We believe that the indication for C1 laminectomy should be judiciously assessed on a case-by-case basis, taking into consideration factors such as the exact location of the lesion and the extent of tonsillar descent. The craniotomy extents employed in our series of PICA-related aneurysms are presented in Figure 5.

#### 4.2.3. Intradural Maneuvers

The CMF approach was originally developed to access midline lesions of the fourth ventricle [38,39]. More recently, its unilateral lateral variant has been increasingly reported as an effective route for treating VA and PICA aneurysms [8,40]. Kawashima et al. emphasized the importance of thoroughly opening the CMF to maximize cerebellar mobility, allowing the tonsil to be elevated substantially and creating a wide operative corridor [8]. Furthermore, Matsushima et al. reported that by almost completely opening the unilateral CMF, a significant retraction of the biventral lobule could be achieved, enabling the surgeon to obtain a wide and safe operative field for vascular cerebellomedullary cistern lesions, much like how opening the Sylvian fissure is extremely useful in supratentorial surgeries [9]. In our experience, achieving such exposure requires not only splitting the CMF but also carefully dissecting the arachnoid around CN IX within the cerebellomedullary cistern. The lateral extent of the CMF is defined by the foramen of Luschka (lateral recess) [41], which is situated between CN IX and the flocculus–choroid plexus complex [10,42,43]. By dissecting the arachnoid between CN IX and this complex, the opening of the CMF can be extended laterally. Furthermore, since the space between CN IX and CN VII/VIII marks the boundary between the cerebellomedullary and cerebellopontine cisterns [10], continuing the arachnoid dissection along CN IX up to its root entry zone allows the CMF opening to connect directly into the cerebellomedullary cistern. Following near-complete opening of the CMF, dissection of the arachnoid between cranial nerves IX and VII/VIII eliminates the remaining attachments of the tonsil, flocculus, choroid plexus, and biventral lobule. As a result, the cerebellum becomes anchored only by its peduncles, thereby achieving maximal mobility. Such a technique is critical not only in cases where the PICA arises high on the VA but also when distal exposure of the VA is required.

#### 4.2.4. Closure

Although the efficacy of collagen matrix dural substitutes in posterior fossa surgery has been documented [44], no dedicated study has evaluated their use specifically in OA–PICA bypass. In our practice, we reconstruct the dura with a collagen matrix in every case, irrespective of whether an OA–PICA bypass is undertaken. The graft is placed both intradurally and extradurally and secured with fibrin sealant. Crucially, our craniotomy technique intentionally avoids violating the mastoid air cells; this preservation of the air-cell barrier, combined with a two-layer collagen matrix duraplasty, appears to act synergistically to minimize cerebrospinal fluid egress. Consistent with this complementary strategy, we have not encountered troublesome subcutaneous cerebrospinal fluid collections. These observations suggest that when the mastoid air cells are left intact, collagen matrix dural closure provides a highly effective seal even in posterior fossa procedures, including OA–PICA bypass.

There are several limitations in this study. First, our findings should be interpreted with caution because of the observational and retrospective study design. Second, selection bias exists. Because this single-center study was performed in a tertiary referral hospital, it was possible that simple aneurysms with a moderate size and a narrow neck were treated by simple endovascular coil embolization in the affiliated hospitals and thus were excluded from our case series. Third, because of the small number of the patient, validity of our treatment strategy and procedures were only descriptive and thus less conclusive, which warrant further investigations.

## 5. Conclusions

Our experience indicates that individualized treatment selection and meticulous operative planning—especially with respect to craniotomy extent and the creation of an optimal operative field—are crucial for the safe and effective management of complex PICA aneurysms. Although we are not able to draw firm conclusions regarding the validity of our treatment strategies due to the limited number of cases, we want to emphasize that the individualized treatment selection should be comprehensively based on both aneurysm-related parameters (location, morphology, and collateral circulation) and patient-specific factors (age, comorbidities, rupture status, and tolerance to antiplatelet therapy).

## Figures and Tables

**Table 1:** Clinical characteristics of 12 patients with PICA-related aneurysms.

Case	Age	Sex	Morphology	Location	Dome Size	Procedure	Antithrombotic	CSF Leak	New Deficits	Hemorrhage	DWI	mRS
1	75	F	saccular (SAH)	VA-PICA	2 mm	OA-PICA & trapping	None	-	-	-	small	0
2	84	F	saccular (SAH)	VA-PICA	8 mm	clipping	None	-	dysphagia	-		4
3	64	F	saccular	VA-PICA	14 mm	OA-PICA & trapping	None	-	Transient dysphagia	-	small	3 Parkinson’s disease
4	78	F	DA	VA (PICA involved)		FD	DAPT		-	-	-	0
5	45	M	DA	VA (PICA involved)		EPAO	DAPT		-	-	-	0
6	66	M	DA	VA (PICA involved)		OA-PICA & trapping	Aspirin	-	-	-	-	0
7	33	M	thrombosed DA	PICA proximal	20 mm	OA-PICA & trapping	Aspirin	-	-	-	-	0
8	67	M	saccular	BA-AICA (AICA-PICA)	7 mm	Hybrid	Aspirin+ Heparin	-	-	+	-	1
9	70	F	saccular	VA-PICA	4.5 mm	clipping	None	-	-	-	-	0
10	57	F	saccular	VA-PICA	3 mm	OA-PICA & trapping	None	-	-	-	moderate	0
11	54	F	saccular	VA-PICA	11 mm	coil embolization	DAPT		-	-	-	4 aneurysmal mass effect
12	44	F	fusiform	PICA origin		EPAO	DAPT		-	-	-	0

Case 3 had an mRS score of 3 due to Parkinson’s disease, but there was no postoperative deterioration. Case 11 had an mRS score of 4, but there was no deterioration compared with the preoperative status.

## Data Availability

The raw data supporting the conclusions of this article will be made available by the authors on request.

## Abbreviations

The following abbreviations are used in this manuscript:

AICA	Anterior inferior cerebellar artery
BA	Basilar artery
CC	Condylar canal
CMC	Cerebellomedullary cistern
CMF	Cerebellomedullary fissure
CN	Cranial nerve
CSF	Cerebrospinal fluid
CT	Computed tomography
DA	Dissecting aneurysm
DAPT	Dual antiplatelet therapy
DWI	Diffusion-weighted imaging
FD	Flow diverter
EVT	Endovascular treatment
ICG	Indocyanine green
INL	Inferior nuchal line
JT	Jugular tubercle
mRS	Modified Rankin Scale
OA	Occipital artery
EPAO	Endovascular parent artery occlusion
PICA	Posterior inferior cerebellar artery
SAH	Subarachnoid hemorrhage
STA-MCA	Superficial temporal artery—middle cerebral artery
VA	Vertebral artery
